# Association between *GSTM1* and *GSTT1* polymorphisms and susceptibility to methamphetamine dependence

**Published:** 2015-01

**Authors:** Mohammad Rashid Khalighinasab, Khyber Saify, Mostafa Saadat

**Affiliations:** Department of Biology, College of Sciences, Shiraz University, Shiraz 71454, Iran

**Keywords:** *GSTM1*, *GSTT1*, Methamphetamine dependence, Polymorphism

## Abstract

Glutathione S-transferases (GSTs; EC: 2.5.1.18) are ubiquitous multifunctional enzymes, which play a key role in cellular detoxification. Functional genetic polymorphisms in genes encoding *GSTM1* (a member of GST class mu; OMIM: 138350), and *GSTT1* (a member of GST class theta; OMIM: 600436) have been well defined. The functional null alleles of GSTM1 and *GSTT1* represent deletions of *GSTM1* and *GSTT1* genes, respectively. The aim of the present study is to investigate the association between *GSTM1* and *GSTT1* polymorphisms and methamphetamine dependence. The present population-based case-control study was performed in Shiraz (southern Iran). In total, 52 methamphetamine dependence (11 females, 41 males) and 635 healthy controls (110 females, 525 males) were included in this study. The genotypes of *GSTM1* and *GSTT1* polymorphisms were determined by PCR. Neither *GSTM1* (OR=0.92, 95% CI: 0.52-1.61, P=0.771) nor *GSTT1* (OR=0.71, 95% CI: 0.33-1.54, P=0.381) null genotypes were significantly associated with risk of methamphetamine dependence. It should be noted that although there was no association between the *GSTM1* null genotype and risk of methamphetamine dependence, in both genders, there was significant interaction between gender and *GSTM1* polymorphism (P=0.029). The combination genotypes of the *GSTM1* and *GSTT1 *polymorphisms revealed that the genotypes of these two polymorphisms had no additive effect in relation to the susceptibility to methamphetamine dependence. The present study revealed that genetic polymorphisms of *GSTT1* and *GSTM1* are not risk factors for methamphetamine dependence.

## INTRODUCTION

Methamphetamine is now one of the major illicit drugs available worldwide [1]. A better understanding of the etiology of methamphetamine dependence is crucial for improving the prevention and treatment of this severe type of drug dependence. Generally it has been well established that drug-dependence disorders are genetically influenced [2]. Several studies have been revealed that methamphetamine induced the oxidative stress [3-9]. Therefore, methamphetamine abuse results in numerous adverse health effects which is associated with oxidative stress, including myocardial infarction, cognitive deficits, and psychiatric disease.

Glutathione S-transferases (GSTs; EC: 2.5.1.18) are ubiquitous multifunctional enzymes, which play a key role in cellular detoxification. Human GSTs are divided into different classes; including mu and theta. Genetic polymorphisms in genes encoding *GSTM1 *(a member of GST class mu; OMIM: 138350), and *GSTT1* (a member of GST class theta; OMIM: 600436) have been well defined [10, 11]. The functional null alleles of *GSTM1* and *GSTT1* represent deletions of *GSTM1* and *GSTT1* genes, respectively. The association studies between these functional polymorphisms and various multifactorial traits such as several types of cancers [10, 12-16], schizophrenia [17, 18], bipolar disease [19], asthma [20, 21], cataract [22, 23], and cardiovascular diseases [24] were conducted. Immunoblot analysis revealed that GSTT1 and GSTM1 were present in brain [25, 26]. There were two studies investigating the association between *GSTM1* and *GSTT1* polymorphisms and risk of methamphetamine dependence [27, 28], with inconsistent results. These facts sufficiently provide us with a theoretical rational to do the present study. The main aim of the present study is to investigate the association between these polymorphisms and susceptibility to methamphetamine abuse.

## MATERIALS AND METHODS

**Participants: **The present study was performed in Shiraz (Fars province, southern Iran). In total, 52 methamphetamine dependence (11 females, 41 males) and 635 healthy controls (110 females, 525 males) were included in this study. The patients were in methadone maintenance for treating methamphetamine dependence and all of them reported methamphetamine as their primary drug of choice. Control individuals were blood donors, who declared that they did not suffer substance abuse. The mean age (SD) of the patients and the controls were 35.0 (8.5) and 33.6 (9.0) years, respectively. There was no statistically significant difference with regard to age (t=1.06, df=685, P=0.288) between the patients and the controls. There was no significant difference between the two study groups for their gender distribution (*x*^2^=0.48, df=1, P=0.486). Considering the high heterogeneity of the Iranian population [29, 30], the participants were selected from Persian Muslims (Caucasians) living in Shiraz (Fars province, southern Iran). Informed consent was obtained from each subject before the study, which was approved by the institutional review board of our university.

At the time of blood donation, a brief questionnaire that ascertained age, dependency to any drug, age at first time used drug, martial status, history of cancers, cataract, and asthma, and history of drug dependency in the first degree relatives was completed. Considering that the polymorphisms of *GSTT1* and *GSTM1* are associated with several types of cancers, asthma, and cataract, the subjects of the both groups had negative history of cancers, asthma, and cataract. 

**Genotyping** **: **Peripheral blood samples were collected from the participants. Genomic DNA was isolated from EDTA treated blood samples. The PCR conditions for determining the genotypes of *GSTT1* and *GSTM1* polymorphisms were the same as that reported previously [14]. Successful amplification with β-globin specific primers confirmed the proper function of the PCR reaction. To test for contamination, negative controls (tubes containing the PCR mixture, without the DNA template) were incubated in every run. Any sample with ambiguous result due to low yield was retested and a random selection of 15% of all samples was repeated. No discrepancies were discovered upon replicate testing.

**Statistical analysis: **The association between the genotypes of the study polymorphisms and methamphetamine dependence risk were assessed by calculating odds ratios (ORs) and 95% confidence intervals (CIs). The reference group consisted of individuals with positive genotypes of *GSTM1* and *GSTT1*. A probability of p<0.05 was considered as statistically significant. 

Using the GPOWER (www.psycho.uni-duesseldorf.de/aap/projects/gpower) software (version 3.1.3), to detect a real difference in allelic frequency with a power of 0.95, α=0.05, *df*=1, Lambda=13.0, and an effect size of 0.2 (small-medium effect); a minimum sample of 325 would be necessary. The present study is more than sufficiently powered with an *N*=687 to detect a small-medium effect in genotype frequency between the two groups.

## RESULTS AND DISCUSSION

Table 1 shows the genotypic prevalence of the study polymorphisms between the cases and healthy controls. The prevalence of *GSTM1* null genotype was 51.9 and 54.0 percent among patients and controls, respectively. Statistical analysis revealed that the null genotype of *GSTM1* was not associated with the risk of methamphetamine abuse (OR=0.92, 95% CI: 0.52-1.61, P=0.771). After stratification of the participants according their genders, we observed the same finding. It should be noted that although there was no association between the *GSTM1* null genotype and risk of methamphetamine dependence, in both genders, there was significant interaction between gender and *GSTM1* polymorphism (P=0.029). 

The frequency of null genotype of *GSTT1* was 15.4 and 20.5 percent in patient and control groups, respectively. Statistical analysis revealed that polymorphism of *GSTT1* was no statistically associated with susceptibility to methamphetamine dependence (OR=0.71, 95% CI: 0.33-1.54, P=0.381) (Table 1). After stratification of the participants according to their genders, the same result was observed. 

**Table 1:** Association between genetic polymorphisms of *GSTM1* and *GSTT1 *and risk of methamphetamine dependence

**Genotypes**	**Patients**	**Controls**	OR*	**95% CI**		**P**
***GSTM1*** ** polymorphism**						
**Both Genders**						
Positive	25	292	1.0	-		-
Null	27	343	0.92	0.52-1.61		0.771
					
**Males**						
Positive	24	246	1.0	-		-
Null	17	279	0.62	0.321.19		0.152
					
**Females**						
Positive	1	46	1.0	-		-
Null	10	64	7.18	0.89-58.1		0.064
					
***GSTT1*** ** polymorphism**						
**Both Genders**						
Positive	44	505	1.0	-		-
Null	8	130	0.71	0.33-1.54		0.381
					
					
**Males**						
Positive	35	411	1.0	-		-
Null	6	114	0.61	0.25-1.50		0.290
					
**Females**						
Positive	9	94	1.0	-		-
Null	2	16	1.30	0.25-6.60		0.747

To investigate whether one null genotype could be compensated by an active genotype for the other isoenzymes in relation to substance abuse, we considered the association between combinations of the genotypes and risk of methamphetamine dependency. The reference group consisted of individuals with “positive genotypes of *GSTM1* and *GSTT1*”. In overall (and also in males), there was no significant association between combined genotypes and susceptibility to methamphetamine abuse (Table 2). There was no linear trend in risk associated with zero, one and two null genotypes (*x*^2^= 0.59; P=0.441).

In the present case-control study, we found that there was no statistically significant association between *GSTM1* polymorphism and risk of methamphetamine dependence. Previously, only one study investigated the association between *GSTM1* polymorphism and susceptibility to methamphetamine abuse in Japan [27]. They reported that the risk of methamphetamine dependence associated with *GSTM1* null genotype was significantly higher only in females than in subjects with the *GSTM1* genotype. Considering that we found that there was significant interaction between gender and *GSTM1 *polymorphism, our present findings were partially consistent with that report. However, this discrepancy might be at least in part interpreted by our small sample size of female cases.

**Table 2:** Associations between combination genotypes of polymorphisms of *GSTM1* and *GSTT1 *and risk of methamphetamine dependence

**Combinations**		**Patients**	**Controls**	OR*	**95% CI**	**P**
**Both genders**						
***GSTM1***	***GSTT1***					
Positive	Positive	20	236	1.0	-	-
Positive	Null	5	56	1.05	0.38-2.93	0.920
Null	Positive	24	269	1.05	0.57-1.95	0.871
Null	Null	3	74	0.48	0.14-1.66	0.244
						
**Males**						
***GSTM1***	***GSTT1***					
Positive	Positive	20	195	1.0	-	-
Positive	Null	4	51	0.76	0.25-2.33	0.765
Null	Positive	15	216	0.67	0.33-1.35	0.273
Null	Null	2	63	0.31	0.07-1.36	0.121

There were two published studies investigating the association between *GSTT1* polymorphism and risk of methamphetamine dependence [27, 28]. Our present finding (no significant association between null genotype of *GSTT1* and susceptibility to methamphetamine dependence) was in agreement with one of them [28].

It is well established that the GSTs are involved in detoxification of a variety of compounds, some of which overlap between these enzymes and some of which are highly specific [31]. Previous studies showed that the null genotypes of *GSTM1* and *GSTT1 *polymorphisms may have additive effect on the risk of multifactorial traits [14, 20]. However, we found that combinations of *GSTT1 *and *GSTM1* polymorphisms are not associated with risk of methamphetamine dependence (Table 2). This finding is not consistent with one of the previous published study [27]. 

We stratified our participants by gender, which reduced sample sizes especially for females; therefore the present analyses on females may have been statistically underpowered. Considering the fact that ethnicity may influence the observed associations in multifactorial diseases, differences between our ethnicity and Japanese ethnicity might be involved. In order to address the involvement of the polymorphisms of *GSTT1* and *GSTM1* on susceptibility to methamphetamine abuse replication of this study in other countries is recommended.

## Notes

**Acknowledgements**: The authors are indebted to the participants for their close cooperation. This study was supported by Shiraz University.
